# Comprehensive proteomic characterization of pulmonary arterial hypertension in Chinese people

**DOI:** 10.3389/fmolb.2025.1652083

**Published:** 2025-08-14

**Authors:** Tianya Liu, Siqi Zhou, Rui Wang, Xiaomei Xu, Fang Gao, Jie Zu, Zhiping Wang

**Affiliations:** ^1^ Department of Anesthesiology, The Affiliated Hospital of Xuzhou Medical University, Xuzhou, China; ^2^ Jiangsu Province Key Laboratory of Anesthesiology, School of Anesthesiology, Xuzhou Medical University, Xuzhou, China; ^3^ Department of Gastroenterology, Nanjing Drum Tower Hospital Clinical College of Jiangsu University, Xuzhou, China; ^4^ Institute of Stroke Center and Department of Neurology, The Affiliated Hospital of Xuzhou Medical University, Xuzhou, China

**Keywords:** PAH, RNA-seq, proteomics, phosphoproteomics, pulmonary vascular remodeling

## Abstract

**Background:**

Pulmonary arterial hypertension (PAH), a serious disease, is characterized by various degrees of pulmonary vascular remodeling, inflammation, and increased vascular resistance, leading to fatalities in patients with severe conditions. However, the molecular mechanisms underlying the pathogenesis of PAH remain incompletely understood.

**Methods:**

RNA sequencing (RNA-seq), 4D label-free proteomics, and phosphoproteomics were employed to detect the levels of mRNA, proteins, and phosphorylation modification in the lung tissues of PAH patients, compared to those in the control group. Parallel reaction monitoring (PRM) was subsequently performed to verify the differentially expressed proteins (DEPs) identified by proteomic profiling.

**Results:**

After data filtering (|log2FoldChange| > 1 and p < 0.05), the PAH group exhibited 967 differentially expressed genes (DEGs), 764 DEPs, and 411 phosphorylated DEPs compared with those of the control group. By integrating transcriptomic and proteomic analyses, 54 proteins were identified with consistent changes at both levels. We analyzed several proteins using PRM, including known candidates such as enolase 1 (ENO1) and chloride intracellular channel 1 (CLIC1), as well as novel proteins such as caveolin-2 (CAV2) and eukaryotic translation initiation factor (EIF2A). Gene Ontology (GO) and Kyoto Encyclopedia of Genes and Genomes (KEGG) analyses of DEPs showed significant enrichment of biological processes associated with inflammatory response, oxidative stress, and tissue remodeling. Phosphorylated DEPs showed significant enrichment in key pathways, including autophagy, apoptosis, and hypoxia inducible factor (HIF) signaling, all of which were closely associated with PAH.

**Conclusion:**

Dysregulated pathways such as autophagy, apoptosis, and HIF-1 signaling, along with altered genes or proteins, contribute to PAH by inducing pulmonary vascular remodeling and chronic vasoconstriction. These findings may facilitate the discovery of novel therapeutic targets and effective treatment strategies for PAH.

## Introduction

Pulmonary vascular remodeling (PAH) is a rarely progressive and incurable disease, which is characterized by elevated pulmonary arterial pressure and vascular resistance, with subsequent ventricular dilatation and right heart failure, eventually leading to death (Hassoun, 2021). Epidemiological data suggest that approximately eight million individuals in China are affected by PAH (Chen et al., 2020). Multiple factors, such as germline mutation, inflammation, pulmonary arterial endothelial cell dysfunction, and metabolic derangements, contribute to the pathogenesis of the disease (Hoeper et al., 2017). These disturbances initiate progressive pulmonary arterial vasoconstriction and vascular remodeling, resulting in abnormal proliferation and apoptosis of the smooth muscle cells. Classic drugs, such as endothelin receptor antagonist, soluble guanylate cyclase agonist, phosphodiesterase type-5 inhibitor, and prostaglandin agonists or analogs, could alleviate the clinical symptoms and improve the prognosis. However, these medications are limited to clinical treatment improvement and cannot solve the fundamental problems, thus potentially allowing for disease progression (Vazquez and Klinger, 2020). Consequently, there is a pressing need to identify novel therapeutic targets and develop more effective treatments for PAH (McGee et al., 2018).

Because of the complicated pathogenesis of PAH, existing pharmacotherapies are inadequate for reversing the pulmonary vascular remodeling or other crucial pathological alterations (Condon et al., 2022). Lung transplantation currently remains the only life-saving therapeutic option for advanced PAH patients despite the 5-year survival rate remaining poor (Farber et al., 2015). Therefore, a deeper understanding of PAH’s underlying mechanisms and identification of novel therapeutic targets are urgently needed.

Multi-omics is a new approach that provides a more comprehensive whole-genome analysis of specific cell types or tissues (Hasin et al., 2017). Transcriptomics focuses on genome-wide RNA expression profiles, proteomics is the large-scale analysis of the entire protein, and phosphoproteomics analysis is used to detect the key post-translational modification during biological processes (Dong and Chen, 2013; Mendes and Dittmar, 2022; Urban, 2022). More importantly, integrated multi-omics approaches, which combine individual omics datasets, are very critical for elucidating the diverse pathological processes in PAH. In this study, we utilized RNA-seq and the 4D label-free approach for proteomics, and phosphoproteomics analyses were used for lung tissues of PAH patients to characterize the molecular signatures. We further combined the results of these dysregulated expressions of genes, proteins, and protein phosphorylation to unveil the potential mechanisms of PAH.

## Materials and methods

### Tissue samples

Lung tissues were obtained from the Wuxi People’s Hospital and the Affiliated Hospital of Xuzhou Medical University from December 2022 to May 2023, and all patients provided their written informed consent. The study protocol was approved by the institutional research ethics committee, and all experimental methods were performed in accordance with the relevant guidelines and regulations. Lung tissues of the PAH group were isolated from the lungs of patients receiving lung transplantation (n = 8), while the control group were undergoing chest surgery (n = 8). PAH was diagnosed in every patient through a clinical method, and the isolated tissue samples were frozen in liquid nitrogen and stored at −80°C for subsequent experiments.

### RNA isolation and RNA-seq

Transcriptome sequencing was performed at Genechem Biotechnology Co., Ltd. (Shanghai, China) following the standard experimental procedures as described (Chen et al., 2021). Total RNA was extracted from tissues using a NEBNext® Ultra II Directional RNA Library Prep Kit (NEB, United States), and 1 μg RNA per sample was used for the RNA sample preparations. The RNA integrity number (RIN) was assessed for all control samples. To ensure the reliability of downstream analyses, only samples with a RIN value >7.0 were included in this study. Then, RNA was fragmented and purified to construct libraries. PCR products were purified, and the library quality was assessed on the Agilent Bioanalyzer 2100 system (Agilent Technologies, CA, United States) according to the manufacturer’s instructions. RNA-seq-based transcriptome profiling was performed by the high-throughput Illumina NovaSeq 6000 sequencing platform (Illumina Technologies, CA, United States), and 150 bp paired-end reads were generated.

### RNA-seq analyses

Reference genome and gene model annotation files were downloaded from genome websites directly. FeatureCounts were used to count the read numbers mapped to each gene and for quantification of the gene expression level. Differential expression analysis between the two groups was performed using the DESeq2 software, which provided statistical routines in digital gene expression data based on the negative binomial distribution. The resulting p-values were adjusted using Benjamini and Hochberg’s approach for controlling the false discovery rate. Genes with an adjusted p-value < 0.05 found by DESeq2 were assigned as differentially expressed. The corrected p-value of 0.05 and |log2(fold change) | were set as the threshold for significant differential expression, and cluster Profiler R package was used for the enrichment analysis.

### Protein extraction and preparation

SDT buffer was added to the tissues, and the lysate was homogenized by an MP automated homogenizer (6.0 M/S, 30 s, twice). The homogenate was sonicated and boiled for 10 min, followed by centrifugation at 14,000 *g* for 15 min. Then, the supernatant was filtered with 0.22-µm filters. The protein was quantified by the BCA Protein Assay Kit (P0012, Beyotime, China) using SDS-PAGE electrophoresis for quality control. The quantitative analysis and SDS-PAGE results confirmed that the samples exhibited high protein quality and sufficient total protein for subsequent experiments. Filter-aided sample preparation (FASP) is a universal method for peptide segments, and the peptide was desalted by a C18 column (Wiśniewski et al., 2009). Similarly, the labeled peptides were combined and desalted using a C18 cartridge, and the mixture was subjected to a High-Select™ Fe-NTA Phosphopeptide Enrichment Kit (Thermo Fisher scientific, A32992). Finally, the eluent was dried down via vacuum centrifugation at 45°C and then dissolved in 0.1% formic acid buffer.

### 4D label-free quantitative proteomics and phosphorylation proteomics

4D label-free quantitative proteomics and phosphorylation proteomics were performed by Genechem Biotechnology Co., Ltd. (Shanghai, China). 4D label-free technology could quantitatively determine the protein levels without complex labeling or processing of samples (Zhao et al., 2020). Before mass spectrometry (MS) identification, the samples were separated using the NanoElute system (Bruker, Bremen, Germany) with a nanoscale flow rate. Then, the samples were analyzed by the timsTOF Pro (Bruker, Bremen, Germany) with the parallel accumulation–serial fragmentation (PASEF) mode. The duration of analysis was 90 min (for phosphorylation analysis, time was 120 min), and the detection model was positive ion. The mass range of the mother ion was 100–1,700 m/z, and the ion mobility started from 0.75 V to 1.4 V⋅s/cm^2^. The ion ramp time was 100 ms, and the utilization rate was 100%. In addition, the capillary voltage was 1,500 V, the drying gas speed was 3 L/min, and the temperature was 180°C. The mass charge ratio of the peptides and peptide fragments was collected according to the following methods: 10 MS/MS scans (total cycle time of 1.16 s), and the charge range was 0–5, the active exclusion was 0.5 min, the scheduling target intensity was 10,000, the intensity threshold was 2,500, and the normalized collision energy was 20–59 eV.

### Protein identification and quantification analysis

Raw files were processed by MaxQuant 1.6.17.0 using the standard settings against the human protein database (Uniprot_HomoSapiens_20337_20220308_swissprot). An initial search was set at 10 ppm, and searches followed an enzymatic cleavage rule of Trypsin/P, which allowed a maximum of two missed cleavage sites and the mass tolerance of 40 ppm for fragment ions. Carbamidomethylation of cysteines was defined as fixed modification, and oxidation of methionines and N-terminal acetylation were defined as variable modifications for searching. The cutoff of the global false discovery rate (FDR) for peptide or protein identification was set at 0.01. Protein abundance was calculated using normalized spectral protein intensity. The significant differentially expressed proteins (DEPs) that were upregulated more than 2-fold or downregulated less than 0.5-fold and had p-value <  0.05 were screened by the UniProt database (https://www.uniprot.org/) for bioinformatics analysis. As previously reported, we only analyzed the protein phosphorylation changes that were higher or lower than its fold change in proteomics (Vasaikar et al., 2019).

### GO annotation

To determine the biological and functional properties of the identified proteins, we employed the hypergeometric test to perform GO enrichment analysis. First, the target protein sequences were aligned to the database using NCBI BLAST+ (ncbi-blast-2.3.0+) on the Linux server, and the top 10 sequences (e-value was less than or equal to 0.001) were kept. Blast2GO was used to select the GO term (database version: go_20190701.obo) of the sequence with the top bit-score and complete the elementary annotation from GO terms for the target protein by the Blast2GO Command Line. In order to improve the efficiency of annotation, InterProScan was used to search the EBI database for conserved motifs matching the protein, and the functional information was added (Quevillon et al., 2005). ANNEX was performed for further annotation information, and the connections between different GO categories were established to improve the accuracy of annotation. Fisher’s exact test was used to enrich the GO terms by comparing the number of DEPs and the total proteins correlated to the GO terms.

### KEGG pathway annotation

KEGG orthology and links annotation software (version number V2.2) was employed for KEGG pathway annotation on the target protein, and KEGG orthology (KO) classified the sequence with information about the pathways automatically. Fisher’s exact test was used to enrich the KEGG terms by comparing the number of DEPs and the total proteins correlated to the KEGG terms.

### Protein validation by PRM

Parallel reaction monitoring (PRM) was performed to determine the levels of the DEPs to verify the proteomic analysis based on the 4D label-free LC-MS/MS. The total protein extraction, enzyme digestion, and desalination methods were the same as described in the previous sample preparation process. Two micrograms of the peptide mixture were loaded onto the C18-reversed phase analytical column (Thermo Fisher Scientific, Acclaim PepMap RSLC 50 um × 15 cm, nanoViper, P/N164943, United States) in buffer A (0.1% formic acid) and separated with a linear gradient of buffer B (80% acetonitrile and 0.1% formic acid) at a flow rate of 300 nL/min. The liquid gradient was as follows: 1–3% B liquid for 0 min–5 min, 3%–28% B liquid for 6 min–45 min, 28%–38% B liquid for 46 min–50 min, 38%–100% B liquid for 51 min–55 min, and 100% B liquid for 56 min–60 min.

Peptide fragmentation and targeted PRM mass spectrometry were performed using a Q Exactive HF-X (Thermo Fisher Scientific, United States) that was coupled to the Easy nLC (Thermo Fisher Scientific, United States) for 60 min in the positive ion mode. Data were acquired using the most abundant precursor ions, with the survey scan range set from 350 to 1,800 m/z for high-energy collisional dissociation (HCD). Survey scans were obtained at a resolution of 60,000 m/z with the AGC target of 3E6 and the maximum injection time of 50 ms. MS2 scans were performed at a resolution of 30,000 m/z for the HCD spectra, with the AGC target of 2E5 and the maximum injection time of 50 ms, and the isolation width was 1.6 m/z. Only the ions with the charge state between 2 and 6 and a minimum intensity of 8E3 were selected for fragmentation. Dynamic exclusion for selected ions was 30 s, and normalized collision energy was 27 eV.

The MS RAW file was analyzed by SpectroDive software, and the database version was uniprot_homo_20230312_20423_9606_swiss_prot. Statistical analysis was performed with SPSS software, and all the results were analyzed using Student’s t-test, with the values being expressed as the means ± standard error. Significant differences were judged by the p-value < 0.05.

## Results

### Screening the differential characteristics in PAH

**FIGURE 1:**
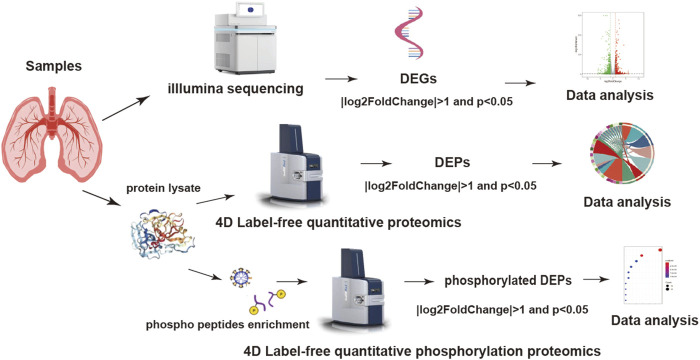
Experimental procedure and multi-omics data analysis workflow.

**FIGURE 2:**
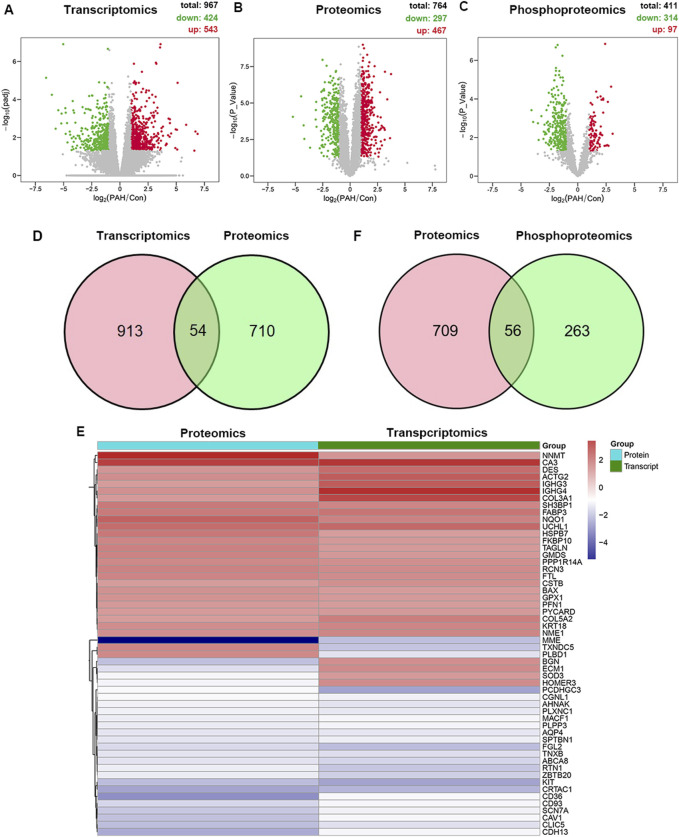
Multi-omics analysis of differentially expressed molecules in the tissues of PAH. **(A)** Volcano plot of transcriptomic changes between the PAH and control groups (n = 8). X-axis: log2 fold change; y-axis: -log10 (p-value); red dots: significantly upregulated genes (p < 0.05, log2FoldChange >1); green dots: downregulated genes (p < 0.05, log2FoldChange < −1). **(B)** Volcano plot of proteomic changes in the control or PAH groups (p < 0.05, |log2FoldChange| > 1). **(C)** Volcano plot of phosphoproteomics changes in the control or PAH groups (p < 0.05, |log2FoldChange| > 1). **(D)** Venny plot of the DEPs from transcriptomics and proteomics. **(E)** Cluster analysis of the overlapping dysregulated genes and proteins. The red and blue boxes represent the upregulated and downregulated proteins, respectively. **(F)** Venny plot of the DEPs from proteomics and phosphoproteomics.

The lung tissue samples from PAH patients or the control patients were analyzed using high-throughput sequencing, including RNA-seq and 4D label-free proteomics, to identify the differentially expressed mRNA, protein, and phosphoprotein. The workflow is presented in Figure 1. Differentially expressed genes (DEGs) were systematically identified with |log2FoldChange|>1 and p < 0.05 as the significant criteria. A total of 424 genes were downregulated, whereas 543 genes were upregulated (Figure 2A; Supplementary Table S1). Subsequently, 4D label-free quantitative proteomics was conducted to investigate the DEPs between the two groups. A total of 4,049 proteins were identified, of which 764 DEPs met the data filtering criteria (|log2FoldChange| >1 and p < 0.05). Among these DEPs, 467 were increased and 297 were reduced (Figure 2B; Supplementary Table S2). In addition, the top 10 upregulated and downregulated proteins in each group were exhibited in detailed heatmaps (Supplementary Figure S1). Those DEPs indicated the dysregulated proteins’ expression during PAH progression. Meanwhile, we discovered 2,197 proteins in phosphoproteome analysis, including 314 downregulated and 97 upregulated phosphoproteins (Figure 2C; Supplementary Table S3). The top 10 most significantly altered phosphoproteins and phosphorylation sites are shown in Supplementary Figure S2.

We integrated transcriptomic and proteomic data and found 54 proteins exhibiting concordant alterations at both mRNA and protein levels, including 48 proteins showing expression trends consistent with their mRNA and six proteins exhibiting opposite trends (Figures 2D, E). Enolase 1 (ENO1), an essential protein for pulmonary artery smooth muscle cell (PASMC) proliferation and de-differentiation, was significantly elevated in our study, which is consistent with the prior reports (Dai et al., 2018). Additionally, we confirmed the altered expressions of several proteins, such as caveolin-1 (CAV1) and chloride intracellular channel protein 1 (CLIC1), which exhibited trends consistent with those in previous studies (Abdul-Salam et al., 2010; Mathew, 2021; Zhou et al., 2022). These pronounced changes confirmed the successful establishment of PAH patient-derived materials. Furthermore, we found previously unreported protein alterations in PAH patients, such as alkaline phosphatase, tissue-nonspecific isozyme (ALPL), membrane metalloendopeptidase (MME), and versican (VCAN). Furthermore, a Venn diagram comparing DEPs and phosphorylated DEPs was constructed (Figure 2F). Overall, multi-omics analysis revealed different characteristics between the two groups.

### Transcriptome profiling between PAH and control patients

**FIGURE 3:**
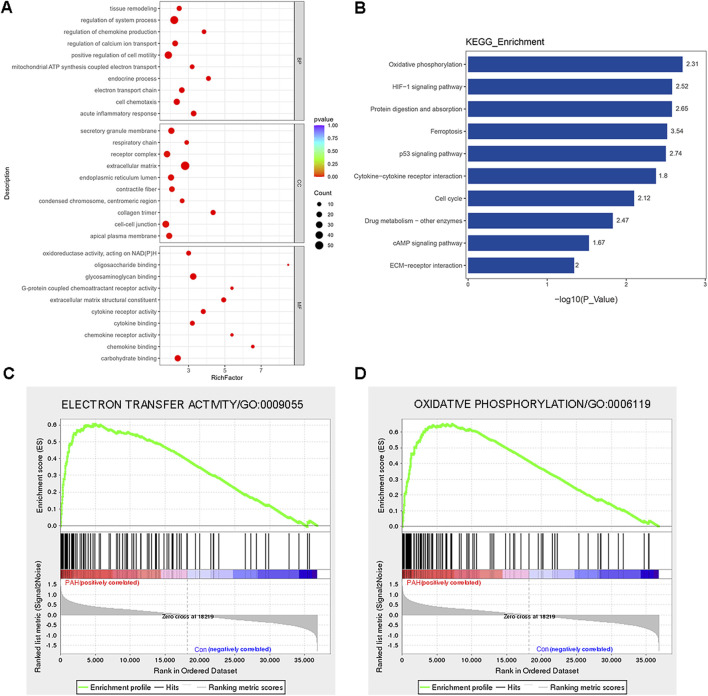
Functional classification based on the pathway enrichment analysis of differentially expressed genes in lung tissues from patients with PAH compared with that of control. **(A)** Go functional classification of DEGs using Fisher’s exact test for biological process (BP), molecular function (MF), and cellular component (CC) categories (|log2FoldChange| > 1, p < 0.01). **(B)** KEGG pathway classification of DEGs using Fisher’s exact test (|log2FoldChange| > 1, p < 0.05). The vertical axis represents the KEGG pathways in each category, and the numbers beside the bars are enrichment factors, which represent the significance and reliability of the proteins enriched in this item. The reliability of proteins in an item was enhanced when the value increased. The horizontal axis shows the -log10 (p-value) of each item. **(C)** GSEA showed enrichment in the electron transfer activity (NES = 1.95, p = 0.008, and FDR = 0.24). **(D)** GSEA showed enrichment in the oxidative phosphorylation (GO: 000619, NES = 1.80, p = 0.018, and FDR = 0.18). Comparison of samples, NES, nominal p-value, and FDR were determined by the GSEA software application, and were indicated within each enrichment plot.

Transcriptomes serve as fundamental regulatory systems in all cellular organisms. To find the possible novel molecular factors or pathways associated with PAH, GO and KEGG enrichment analyses were performed to analyze DEGs. The analysis of GO terms was categorized into three parts, namely, biological process (BP), molecular function (MF), and cellular component (CC). DEG enrichment analysis revealed predominant involvement in the endocrine process, acute inflammatory response, regulation of chemokine production, electron transport chain, tissue remodeling, and NADH dehydrogenase activity (Figure 3A). KEGG pathway analyses revealed that DEGs were highly enriched in oxidative phosphorylation, hypoxia inducible factor-1 (HIF-1) signaling pathway, ferroptosis, and p53 signaling pathway in PAH patients (Figure 3B). In particular, the most enriched BPs among DEGs were related to energy and metabolism, including the electron transport chain, oxidative phosphorylation, and purine ribonucleoside triphosphate metabolic processes. For the downregulated DEGs, the enriched BPs included cell–cell adhesion via plasma-membrane adhesion molecules and neutrophil activation involved in the immune response. For MF, the most significantly enriched GO terms for upregulated and downregulated DEGs were the extracellular matrix (ECM) structural constituent and the cytokine receptor activity, respectively. In CC, extracellular matrix and secretory granule membrane were the most significantly enriched GO terms (Supplementary Figure S3A–C). The top three KEGG pathways among the upregulated DEGs were oxidative phosphorylation, p53 signaling pathway, and cell cycle, and those among the downregulated DEGs were cytokine–cytokine receptor interaction, cAMP signaling pathway, and cell adhesion molecules (Supplementary Figure S3D).

To gain insight into the function of the detected genes, gene set enrichment analysis (GSEA) was conducted. Based on the absolute values of the normalized enrichment score (NES) > 1, nominal p value < 0.05, and FDR < 0.25, the signaling pathways of electron transfer activity (GO: 0009055, NES = 1.95, p = 0.008, and FDR = 0.24) and oxidative phosphorylation (GO: 000619, NES = 1.80, p = 0.018, and FDR = 0.18) were significantly enriched (Figures 2C, D). Detailed information regarding genes contributing to the enriched terms is provided in the Supplementary Table S4. In summary, the DEGs were primarily enriched in inflammation, oxidative stress, metabolism, and several other important signaling pathways of PAH patients.

### Proteomic profiling between PAH and control patients

**FIGURE 4:**
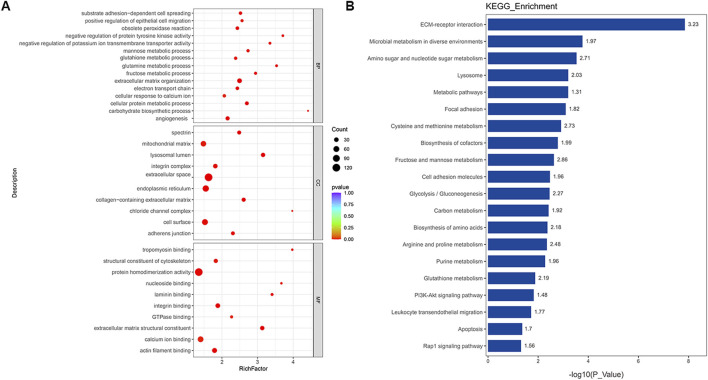
Functional classification based on the pathway enrichment analysis of different proteins in PAH samples compared to that in controls. **(A)** Enriched GO terms in the biological process (BP), molecular function (MF), and cellular compartment (CC) (p < 0.01). **(B)** KEGG pathway enrichment analysis of significantly altered proteins (p < 0.05). The horizontal axis represents the significance of each pathway in the form of -log10 (p-value). The numbers beside the bars are enrichment factors for each pathway.

To elucidate the functional impact of protein networks and pathways in PAH, 764 DEPs were subjected to GO and KEGG analyses of PAH patients. BP terms enrichment analysis revealed the dysfunction in extracellular matrix organization, angiogenesis, electron transport chain, and metabolism disorder, such as the metabolism of glutamine, fructose, and other substances. It was observed that the term “electron transport chain” appeared in both transcriptomics and proteomics results, which meant that this biological process played a significant role in the progression of PAH. For MF, the most significantly enriched GO terms for DEPs were “binding” and “extracellular matrix structural constituent,” and the CC terms revealed that lysosomal lumen and the adherens junction were significantly enriched (Figure 4A). Similarly, the top 20 KEGG pathway analyses suggested the involvement of ECM–receptor interaction, lysosome, PI3K-AKT signaling pathway, and Rap1 signaling pathway in the progression of PAH (Figure 4B). The most significantly altered terms were enriched in metabolic pathways, including the metabolism of amino acids, purine, glutathione, and glycolysis. Several key proteins were implicated in regulating disease progression through various pathways. Detailed information on the proteins contributing to each enriched item is provided in the Supplementary Table S5. In summary, alterations in DEPs related to signaling pathways, metabolism, and dysfunctional cellular processes might be central to PAH pathogenesis.

### Functional analysis of phosphorylated DEPs between PAH and control patients

**FIGURE 5:**
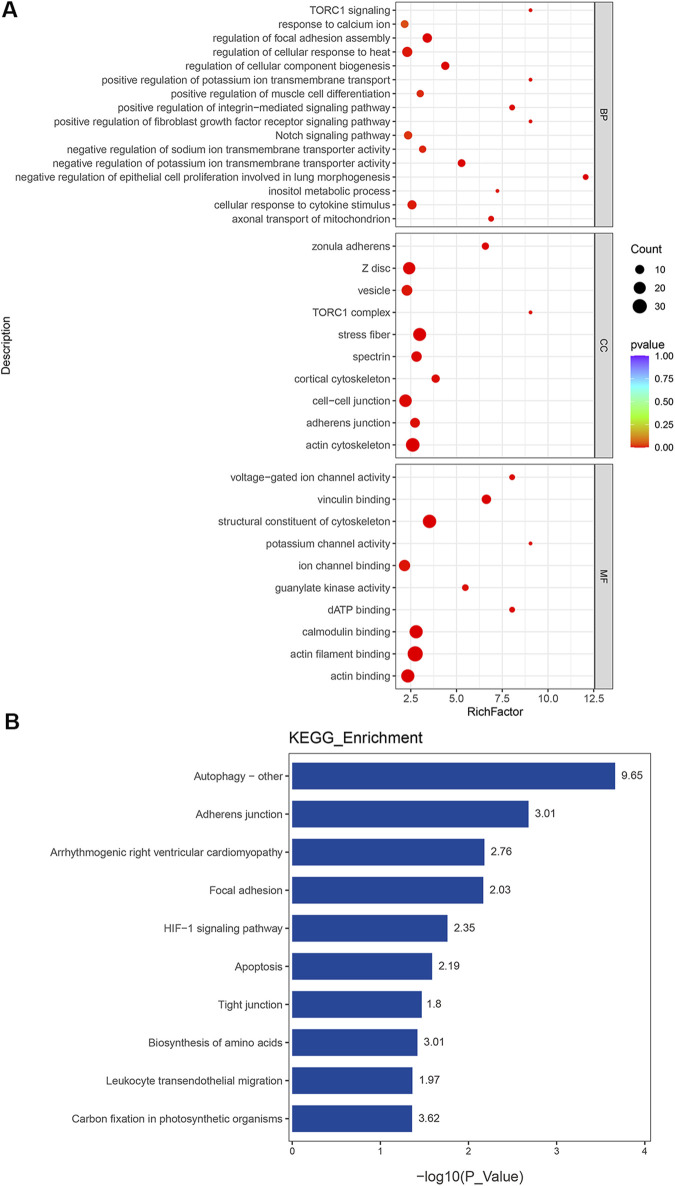
Functional classification based on the pathway enrichment analysis of differentially expressed phosphoproteins in PAH compared to those in controls. **(A)** GO functional classification of differentially expressed phosphoproteins (p < 0.01). **(B)** Rich factor of significant KEGG pathways (p < 0.05).

Protein phosphorylation serves as a critical post-translational modification, dynamically controlling protein activity in diverse cellular processes. The GO and KEGG enrichment analyses of these 411 phosphorylated DEPs proved to be essential for understanding their functional roles in PAH. It should be emphasized that protein entries were excluded if the fold change in phosphorylation levels was less than the fold change observed in the total protein expression before enrichment analysis. The GO enrichment analysis showed that the phosphorylated DEPs were most significantly enriched in all the reference sets (Figure 5A). These phosphorylated proteins participated in various cellular processes, such as epithelial cell proliferation involved in lung morphogenesis, negative regulation of potassium ion transmembrane transporter activity, cellular response to cytokine stimulus, response to calcium ions, and the notch signaling pathway in BP. For MF, the most significantly enriched GO terms were “binding” and structural constituent of cytoskeleton, voltage-gated ion channel activity, and potassium channel activity. Cell–cell junction and stress fiber were the enriched CC terms. Meanwhile, the KEGG enrichment analysis revealed that the most significant enriched terms were autophagy, HIF-1 signaling pathway, tight junction, and leukocyte transendothelial migration (Figure 5B). Detailed descriptions of the enriched protein entries are available in the Supplementary Table S6. Among these pathways, the HIF-1 signaling pathway was observed in both transcriptomic and proteomic KEGG analyses, suggesting its potential critical role in the pathogenic progression of PAH. In summary, phosphorylated DEPs had greater contributions to key cellular processes, including ion channel regulation and metabolism, which were tightly associated with PAH evolution.

### Verification of the key proteins

To further validate the proteomics findings, PRM was carried out to quantify the expression of the selected proteins. We prioritized some proteins for experimental validation to confirm the success of the PAH model, such as ENO1 and chloride intracellular channel-1 (CLIC1). Our selection also covered novel protein candidates that had not been reported previously as clinically relevant, such as CAV2 and EIF2A. Moreover, desmin (DES) and sodium channel protein type 7 subunit alpha (SCN7A) with concordant multi-omics expression profiles were prioritized for further analysis. Additionally, proteins linked to significantly enriched GO terms or KEGG pathways were verified. For example, dematin actin binding protein (DMTN) was enriched in the GO term “positive regulation of epithelial cell migration.”

**FIGURE 6:**
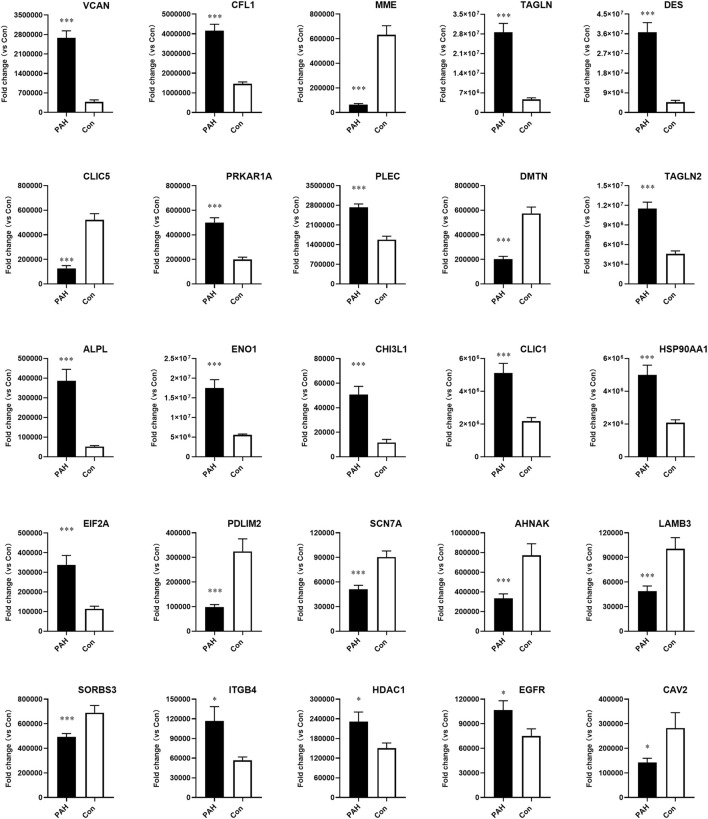
Validation of candidate protein expression using parallel reaction monitoring (PRM) analysis in PAH samples compared to controls. VACN, CFL1, MME, TAGLN, DES, CLIC5, PRKAR1A, PLEC, DMTN, TAGLN2, ALPL, ENO1, CHI3L1, CLIC1, HSP90AA1, EIF2A, PDLIM2, SCN7A, AHNAK, LAMB3, SORBS3, ITGB4, HDAC1, EGFR, and CAV2 were detected in lung tissues from PAH patients (n = 8, *p < 0.05, and ***p < 0.001).

Similarly to prior proteomics research, there are some discrepancies between the two approaches (Liao et al., 2021; Wang et al., 2021). Relative quantification demonstrated concordant expression levels for 25 proteins between 4D label-free and PRM analyses, whereas five proteins displayed no statistically significant differences (Figure 6). VCAN, MME, CLIC1, and other selected proteins exhibited statistically significant fold changes that were consistent across the two methods, and the detailed information of the proteins verified by PRM is summarized in Supplementary Table S7.

## Discussion

Current pharmacotherapies can improve the clinical symptoms of PAH; however, lung transplantation remains the only definitive intervention for advanced-stage PAH. In this study, we identified several dysregulated genes or proteins from the lung tissues of PAH patients using RNA-seq and 4D label-free technology. By integrating multi-omics profiling, we investigated the complex pathogenic mechanisms underlying PAH. Furthermore, GO and KEGG enrichment analyses of significantly altered proteins established PAH molecular profiles, revealing novel signaling pathway dysregulations that provide insights into the disease pathophysiology and potential therapeutic targets.

Transcriptomic analysis revealed 543 significantly upregulated and 424 downregulated genes. Several genes, such as ENO1 and epidermal growth factor receptor (EGFR), demonstrated alterations consistent with previous research (Dai et al., 2018; Yu et al., 2019). The dysregulation of these genes exhibited strong mechanistic links to PAH. GO enrichment analysis of DEGs showed significant enrichment of BPs associated with ‌inflammatory response and oxidative stress, tissue remodeling, such as cell chemotaxis, acute inflammatory response, and positive regulation of cell motility. Oxidative phosphorylation and the HIF-1 signaling pathway were ranked as the top significantly regulated KEGG pathways in the PAH group. It was previously known that PAH caused higher oxidative stress and a stronger inflammatory response in epithelial cells, and our experiments reproduced these results (Shen et al., 2024). Chronic hypoxia was one of the major contributing factors to the development of the pathological condition in PAH (Ahmed et al., 2024). HIF signaling had been reported to be a crucial pathway in chronic hypoxia that promoted PAH development through vascular remodeling and pulmonary vascular dysfunction (Pullamsetti et al., 2020; Mitra et al., 2024). Our phosphoproteomics results also demonstrated the HIF-1 signaling pathway in subsequent analyses. These results provided clear evidence that HIF signaling was a viable strategy for clinical translation. GSEA showed significant enrichment of oxidative phosphorylation pathways in PAH, and antioxidant treatment might serve as the other potential therapeutic option for PAH (Xu et al., 2022; Pokharel et al., 2023). The pulmonary vasculature in PAH exhibited cancer-like molecular features, including dysregulated genes linked to neutrophil activation, chemokine signaling, and calcium homeostasis, which may be either the cause or consequence of PAH (Guignabert et al., 2013; Antigny, 2022). Ferroptosis is a newly identified, iron-dependent form of regulated cell death that plays critical roles in various organ injuries and cancers (Zhang and Liu, 2021). Our data indicated that DEGs in ferroptosis and p53 signaling were correlated with PAH. In conclusion, the candidate genes and enriched pathways from the transcriptomic data need further experimental validation.

It was well known that proteins that were expressed by genes after transcription were the true functional executors and key determinants of cell processes. We further performed 4D label-free proteomic analysis to screen protein expression profiles in PAH. Our results revealed 764 significantly altered proteins, comprising 467 upregulated and 297 downregulated proteins. Most proteins showed functional enrichment in autophagy regulation, angiogenesis, metabolism, ion channels, and acute inflammatory response. GO analysis suggested a pronounced contributory role of angiogenesis, electron transport chain, ion channel, and metabolic regulation. The KEGG pathway analysis showed significant enrichment in apoptosis, and PI3K-AKT and Rap1 signaling pathways, as well as in the metabolic pathways and secondary metabolites’ biosynthesis pathway. Previous studies suggested that the inhibition of the PI3K-AKT pathway might attenuate PAH development, and this pathway was also implicated in autophagy regulation (Zhang et al., 2021; Zhou et al., 2024). Our findings aligned with prior omics-based studies. The Rap1 signaling pathway was involved in various BPs, including cell proliferation and vascular dysfunction (Chrzanowska-Wodnicka, 2013). However, the roles of certain dysregulated pathways, particularly Rap1 signaling, in PAH pathogenesis remain incompletely understood and require further investigation. In addition, our proteomic analysis discovered several novel proteins, such as CAV2 and chloride intracellular channel 5 (CLIC5), which were enriched in multiple critical pathways. No prior omics investigations have reported these proteins. CAV2, a member of the caveolin gene family, was ubiquitously expressed in most cell types and regulated critical cellular processes, including cell migration, metastasis, angiogenesis, and drug resistance (Liu et al., 2018; He et al., 2023). Similarly, its homolog CAV1 promoted PAH progression by impairing endothelial cell proliferation and migration, reducing cytoskeletal stress fibers, and stimulating neointima formation (Han et al., 2016; Gairhe et al., 2021). Although proteomics and PRM demonstrated significant CAV2 downregulation, the functional relevance of this protein in PAH pathogenesis remains unknown. Chloride channels such as CLIC1 and CLIC4 were overexpressed in PAH and contributed to endothelial mitochondrial function and energy metabolism (Alzaydi et al., 2023; Jiang et al., 2023). CLIC5 encoded actin-binding cytoskeletal protein and had been implicated in many human diseases (Huang et al., 2023). Enrichment analysis suggested CLIC5 might participate in peroxidase-related pathways and is associated with PAH in the same way.

To gain deeper insights into PAH pathogenesis, we integrated transcriptomic and proteomic analyses and found 54 genes with concordant expression alterations. This multi-omics approach could overcome the limitations inherent to single-omics analyses (Jiang et al., 2023). We noted that the representative protein DES and MME had the same tendency both in the mRNA and the protein levels. As the principal intermediate filament subunit in all muscle types, DES maintains PASMC homeostasis, and its dysregulation might compromise this critical balance (Paulin and Li, 2004; Agnetti et al., 2022). MME, also known as neprilysin, was responsible for the catalytic inactivation of multiple peptides, including endothelin, and could attenuate the progression of certain human cancers (Cheng et al., 2020; Ding et al., 2023). These proteins potentially modulate signaling pathways in endothelial dysfunction, and further investigation of their roles could advance our understanding of PAH pathogenesis.

Phosphorylation modification was often referred to as the functional “switch” of proteins and directly contributed to PAH development. To enhance phosphosite identification accuracy, we implemented phosphoproteomic background subtraction with blank controls to eliminate nonspecific binding signals. This analytical approach significantly reduced false-positive phosphopeptide identifications and improved the dataset’s reliability. Subsequent profiling of PAH lung tissues identified 97 upregulated and 314 downregulated phosphoproteins under stringent criteria. Importantly, the volcano plot represented the statistical analysis of phosphorylation site changes, whereas each protein may contain multiple phosphorylation sites. Thus, the total number of phosphorylated DEPs in the Venn diagram was 319. GO term analysis revealed significant enrichment in epithelial cell proliferation, potassium ion transmembrane transport, and the notch signaling pathway, along with phosphorylation of key proteins in each term. These phosphorylation proteins included DES, EGFR, VCAN, and neural precursor cell-expressed developmentally downregulated 4-like (NEDD4L). Aberrant expression of these phosphoproteins disrupted proliferation homeostasis and contributed to pulmonary disorder development (Shan et al., 2021; Li et al., 2022). Our preliminary data confirmed that NEDD4L promotes PASMC proliferation, contributing to PAH pathogenesis (Wang et al., 2024). Taken together, these findings were consistent with PAH pathogenesis, which was characterized by dysregulated proliferation across all vascular cell types, including endothelial cells, smooth muscle cells, and fibroblasts (Rafikova et al., 2019). Meanwhile, the KEGG pathway analysis of phosphoproteins uncovered a striking enrichment in autophagy, apoptosis, and HIF-1 signaling pathway. The representative proteins included the mammalian target of rapamycin (mTOR), ENO1, DES, EGFR, and integrin subunit beta 4 (ITGB4). mTOR was a highly important protein kinase that responded to the cellular and extracellular signals (Saxton and Sabatini, 2017). Its phosphorylation contributed to the proliferation, migration, and gene regulation in PASMCs and endothelial cells, leading to pulmonary vascular remodeling and chronic vasoconstriction (Babicheva et al., 2021; Szwed et al., 2021). Our multi-omics analysis revealed that ENO1 was a previously unrecognized essential protein in PAH and participated in many pathways. Given the limited understanding of its role, further investigation into its mechanisms could lead to new therapeutic opportunities.

This study offers the first comprehensive multi-omics integration of PAH lung tissue profiles. However, several limitations should be acknowledged. As the PAH patients were under medical treatment, the observed proteomic alterations may partially reflect drug-induced effects rather than disease-specific changes. Nevertheless, human specimens remain invaluable for PAH research. Future studies should combine *in vitro* models and paired tissue–blood analyses to dissect these confounding factors and elucidate the intrinsic pathophysiology of PAH. Another limitation was our inability to detect alterations in key PAH-associated proteins, such as bone morphogenetic protein receptor type II (BMPR2), STAT3, and phosphor-STAT3 (Balistrieri et al., 2023). This might be due to their lower expression or the failure to meet our fold change thresholds. Combined transcriptomic–proteomic profiling uncovered multi-layer protein regulation, whereas phosphoproteomics included protein modifications (Buccitelli and Selbach, 2020). Given the limited sample size, our findings remain preliminary and require further validation in larger, independent patient cohorts. Collectively, these results provided a sophisticated framework for understanding PAH pathogenesis by integrating multiple layers of molecular regulation.

## Conclusion

In summary, in this study, we conducted an integrative multi-omics analysis of clinical lung tissues, combining transcriptomic, proteomic, and phosphoproteomic data to elucidate the molecular mechanisms underlying PAH pathogenesis. Our multi-omics analyses revealed the comprehensive profiles of dysregulated proteins and pathways, including autophagy, apoptosis, and HIF-1 signaling, thus advancing the molecular understanding of PAH pathogenesis. Further investigation of mRNA, protein, and phosphoprotein alterations may reveal novel therapeutic targets and intervention strategies for PAH.

## Data Availability

The datasets presented in this study can be found in online repositories. The names of the repository/repositories and accession number(s) can be found below: https://www.ncbi.nlm.nih.gov/, https://www.ncbi.nlm.nih.gov/geo/query/acc.cgi?acc=GSE272776.

## Glossary

PAH: Pulmonary arterial hypertension
GO: Gene Ontology
KEGG: Kyoto Encyclopedia of Genes and Genomes
PRM: Parallel reaction monitoring
ENO1: Enolase 1
PASMCs: Pulmonary artery smooth muscle cells
CAV1: Caveolin-1
CAV2: Caveolin-2
CLIC1: Chloride intracellular channel protein 1
CLIC5: Chloride intracellular channel protein 5
MME: Membrane metalloendopeptidase
NEDD4L: Neural precursor cell-expressed developmentally downregulated 4-like
VCAN: Versican
DES: Desmin
TAGLN: Transgelin
CHI3L1: Chitinase-3-like protein 1
DMTN: Dematin actin binding protein
