# Supplementary material for: Comprehensive proteomic characterization of pulmonary arterial hypertension in Chinese people
Source: Front Mol Biosci. 2025 Aug 14;12:1652083. doi: 10.3389/fmolb.2025.1652083 (PMC12391885; doi:10.3389/fmolb.2025.1652083)
Supplement: Supplementary file 3 [file Table7.docx]

**Supplementary Information**

**Integration of transcriptomics, proteomics, phosphoproteomics analysis for characterization of pulmonary arterial hypertension in Chinese people**

Tianya Liu^1, 2†^, Siqi Zhou^3†^, Rui Wang^1^, Xiaomei Xu^1^, Fang Gao^1^, Jie Zu^4^, Zhiping Wang^1, 2*^

Author details

1Department of Anesthesiology, the Affiliated Hospital of Xuzhou Medical University, Xuzhou, Jiangsu, China

2Jiangsu Province Key Laboratory of Anesthesiology, School of Anesthesiology, Xuzhou Medical University, Jiangsu, China

3Department of Gastroenterology, Nanjing Drum Tower Hospital Clinical College of Jiangsu University, Jiangsu, China

4Institute of Stroke Center and Department of Neurology, the Affiliated Hospital of Xuzhou Medical University, Xuzhou, Jiangsu, China

†Tianya Liu and Siqi Zhou contributed equally to this work.

*Correspondence: Zhiping Wang, zhpsqxt@126.com


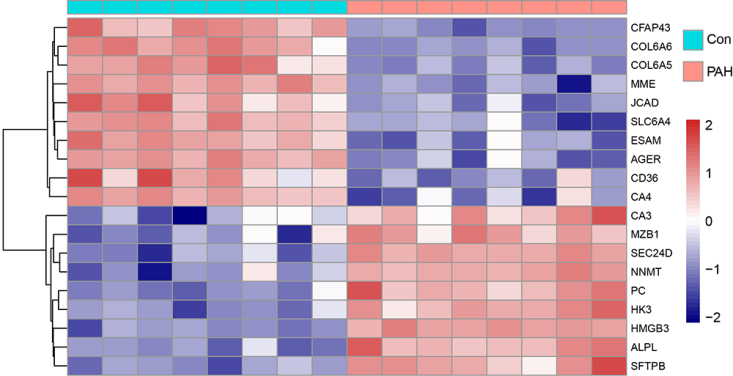


FIGURE S1. Heatmap analysis of the top 10 most significantly altered proteins in pulmonary arterial hypertension (PAH) versus control groups (n=8). The panel displays both markedly upregulated (red) and downregulated (blue) proteins.


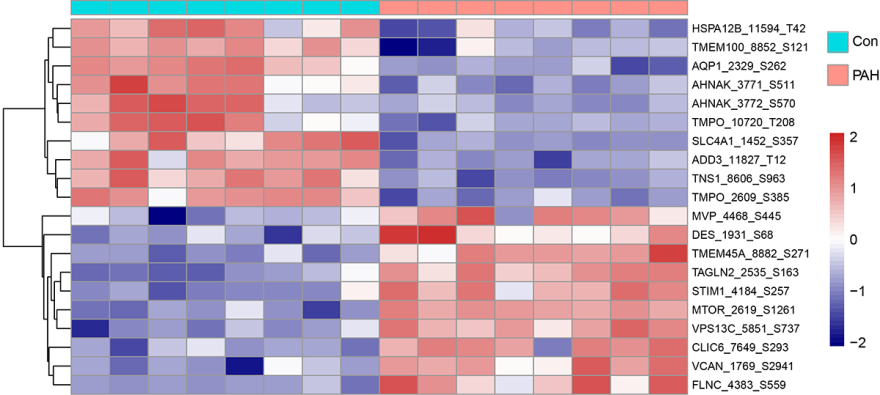
FIGURE S2. Heatmap analysis of the top 10 most significantly altered phosphoproteins in PAH versus controls (n=8). The analysis highlights both markedly upregulated (red) and downregulated (blue) phosphoproteins and their phosphorylation sites.


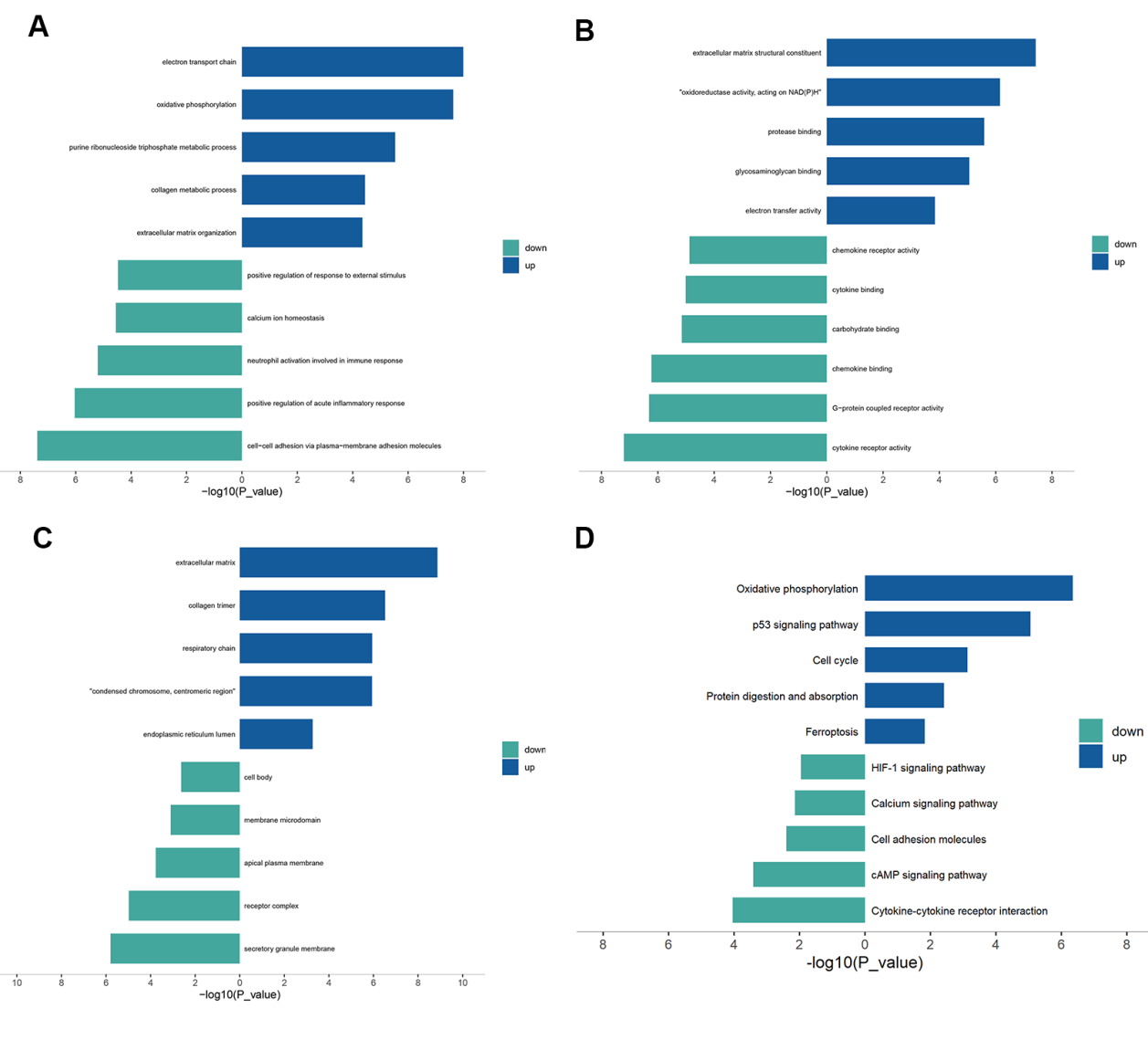


FIGURE S3. Functional enrichment analysis of differentially expressed genes in PAH versus controls. (A-C) Gene Ontology (GO) classification of biological processes, molecular functions, and cellular components for significantly regulated genes (log2FoldChange > 1, p-value <0.01). (D) KEGG pathway analysis of dysregulated pathways (log2FoldChange > 1, p-value <0.05).

**List of includes:**

Supplementary Table S1. 967 differentially expressed genes in lung tissues of patients with pulmonary hypertension (PAH) as compared with that of control group (all P < 0.05).

Supplementary Table S2. 764 differentially expressed proteins in lung tissues of patients with pulmonary hypertension (PAH) as compared with that of control group.

Supplementary Table S3. 411 differentially expressed phosphorylated proteins in lung tissues of patients with pulmonary hypertension (PAH) as compared with that of control group.

Supplementary Table S4. The detailed information of the significant GO and KEGG pathways of differential expressed genes in lung tissues of patients with pulmonary hypertension (PAH).

Supplementary Table S5. Biological functions of proteins differentially expressed in lung tissues of patients with pulmonary hypertension (PAH).

Supplementary Table S6. Biological functions of phosphorylated proteins differentially expressed in lung tissues of patients with pulmonary hypertension (PAH).

Supplementary Table S7. Parallel reaction monitoring validation of proteins with significant difference in 4D Label-free quantitative proteomics.

Table S7. Parallel reaction monitoring validation of proteins with significant difference in 4D Label-free quantitative proteomics (LC-MS/MS)

| Protein Name | Protein  Accessions | Gene Name | Ratio of PAH/Con with PRM | p-value of PRM | Ratio of PAH/Con with proteomics | p-value of proteomics |
| --- | --- | --- | --- | --- | --- | --- |
| Versican core protein | P13611 | VCAN | 7.04 | 4.17E-07 | 2.28 | 5.57E-04 |
| Cofilin-1 | P23528 | CFL1 | 2.85 | 1.44E-06 | 1.71 | 1.76E-04 |
| Neprilysin | P08473 | MME | 0.10 | 2.42E-06 | 0.03 | 8.81E-05 |
| Transgelin | Q01995 | TAGLN | 6.13 | 3.31E-06 | 2.91 | 1.58E-04 |
| Desmin | P17661 | DES | 7.84 | 4.46E-06 | 2.22 | 8.32E-04 |
| Chloride intracellular channel protein 5 | Q9NZA1 | CLIC5 | 0.24 | 4.64E-06 | 0.25 | 5.41E-04 |
| cAMP-dependent protein kinase type I-alpha regulatory subunit | P10644 | PRKAR1A | 2.50 | 5.12E-06 | 1.19 | 3.42E-02 |
| Plectin | Q15149 | PLEC | 1.73 | 1.18E-05 | 0.78 | 1.04E-03 |
| Dematin | Q08495 | DMTN | 0.35 | 1.30E-05 | 0.18 | 1.72E-04 |
| Transgelin-2 | P37802 | TAGLN2 | 2.51 | 1.50E-05 | 1.92 | 1.97E-06 |
| Alkaline phosphatase, tissue-nonspecific isozyme | P05186 | ALPL | 7.36 | 6.26E-05 | 12.48 | 2.57E-03 |
| Alpha-enolase | P06733 | ENO1 | 3.15 | 6.92E-05 | 2.52 | 7.08E-06 |
| Chitinase-3-like protein 1 | P36222 | CHI3L1 | 4.37 | 7.37E-05 | 3.42 | 1.65E-02 |
| Chloride intracellular channel protein 1 | O00299 | CLIC1 | 2.35 | 3.39E-04 | 2.12 | 1.11E-05 |
| Heat shock protein HSP 90-alpha | P07900 | HSP90AA1 | 2.39 | 3.89E-04 | 1.86 | 5.74E-06 |
| Eukaryotic translation initiation factor 2A | Q9BY44 | EIF2A | 2.97 | 4.89E-04 | 4.49 | 6.34E-05 |
| PDZ and LIM domain protein 2 | Q96JY6 | PDLIM2 | 0.30 | 6.14E-04 | 0.32 | 2.55E-05 |
| Sodium channel protein type 7 subunit alpha | Q01118 | SCN7A | 0.56 | 6.17E-04 | 0.28 | 9.97E-05 |
| Neuroblast differentiation-associated protein AHNAK | Q09666 | AHNAK | 0.43 | 3.37E-03 | 0.46 | 4.92E-04 |
| Laminin subunit beta-3 | Q13751 | LAMB3 | 0.48 | 4.14E-03 | 0.20 | 4.55E-06 |
| Vinexin | O60504 | SORBS3 | 0.71 | 9.62E-03 | 0.33 | 3.61E-06 |
| Integrin beta-4 | P16144 | ITGB4 | 2.06 | 1.74E-02 | 0.76 | 5.11E-02 |

| Protein Name | Protein  Accessions | Gene Name | Ratio of PAH/Con with PRM | p-value of PRM | Ratio of PAH/Con with proteomics | p-value of proteomics |
| --- | --- | --- | --- | --- | --- | --- |
| Histone deacetylase 1 | Q13547 | HDAC1 | 1.54 | 3.01E-02 | 0.77 | 1.62E-03 |
| Epidermal growth factor receptor | P00533 | EGFR | 1.39 | 4.37E-02 | 0.65 | 5.30E-03 |
| Caveolin-2 | P51636 | CAV2 | 0.56 | 4.85E-02 | 0.39 | 9.88E-05 |
| LIM and calponin homology domains-containing protein 1 | Q9UPQ0 | LIMCH1 | 0.70 | 8.08E-02 | 0.32 | 5.17E-05 |
| Extracellular superoxide dismutase [Cu-Zn] | P08294 | SOD3 | 1.29 | 8.83E-02 | 0.48 | 6.03E-04 |
| Microtubule-associated protein 4 | P27816 | MAP4 | 0.71 | 1.22E-01 | 0.78 | 1.19E-02 |
| Alpha-adducin | P35611 | ADD1 | 0.93 | 6.31E-01 | 0.40 | 7.37E-08 |
| Catenin beta-1 | P35222 | CTNNB1 | 1.02 | 8.71E-01 | 0.51 | 1.30E-04 |
